# MzrA: a novel modulator of the EnvZ/OmpR two-component regulon

**DOI:** 10.1111/j.1365-2958.2009.06728.x

**Published:** 2009-05-26

**Authors:** Henri Gerken, Emily S Charlson, Elisha M Cicirelli, Linda J Kenney, Rajeev Misra

**Affiliations:** 1 School of Life Sciences, Arizona State UniversityTempe, AZ 85287-4501, USA; 2 School of Medicine, University of PennsylvaniaPhiladelphia, PA 19104, USA; 3 Department of Microbiology and Immunology, University of Illinois at ChicagoChicago, IL 60612, USA

## Abstract

Analysis of suppressors that alleviate the acute envelope stress phenotype of a Δ*bamB*Δ*degP* strain of *Escherichia coli* identified a novel protein MzrA and pleiotropic *envZ* mutations. Genetic evidence shows that overexpression of MzrA – formerly known as YqjB and EcfM – modulates the activity of EnvZ/OmpR similarly to pleiotropic EnvZ mutants and alter porin expression. However, porin expression in strains devoid of MzrA or overexpressing it is still sensitive to medium osmolarity, pH and procaine, all of which modulate EnvZ/OmpR activities. Thus, MzrA appears to alter the output of the EnvZ/OmpR system but not its ability to receive and respond to various environmental signals. Localization and topology experiments indicate that MzrA is a type II membrane protein, with its N-terminus exposed in the cytoplasm and C-terminus in the periplasm. Bacterial two-hybrid experiments determined that MzrA specifically interacts with EnvZ but not with OmpR or the related membrane sensor kinase, CpxA. This and additional genetic and biochemical evidence suggest that the interaction of MzrA with EnvZ would either enhance EnvZ's kinase activity or reduce its phosphatase activity, thus elevating the steady state levels of OmpR∼P. Furthermore, our data show that MzrA links the two-component envelope stress response regulators, CpxA/CpxR and EnvZ/OmpR.

## Introduction

The outer membrane of Gram-negative bacteria is the first defence barrier between the often hostile milieu in which the bacteria live and their intracellular contents. Consequently, integrity of the outer membrane is of utmost importance for bacterial survival and pathogenesis. A unique glycolipid, lipopolysaccharide, coats the outer leaflet of the outer membrane, while phospholipids are partitioned exclusively in the inner leaflet of the outer membrane (Nikaido and Vaara, 1985). The outer membrane is also home to β-barrel-forming outer membrane proteins (OMPs) as well as lipoproteins (Nikaido and Vaara, 1985).

The regulation of β-barrel OMP synthesis has been extensively studied. In particular, the EnvZ/OmpR-mediated regulation of OmpF and OmpC, the two major pore-forming OMPs, has been a paradigm for understanding gene regulation by two-component signal transduction systems (for reviews, see Hoch and Silhavy, 1995). EnvZ is a membrane-bound sensor kinase, although the signal it senses is not known. OmpR is a cytosolic response regulator, which binds to the promoter region of the porin genes. Changes in medium osmolarity profoundly affect OmpF and OmpC expression: OmpC is preferentially expressed in high osmolarity, whereas OmpF expression is favoured in low osmolarity (van Alphen and Lugtenberg, 1977). Although the level of OmpR∼P *in vivo* has not been determined, it is presumed that the [OmpR∼P] increases with increasing osmolarity, promoting activation and then repression of *ompF* and activation of *ompC*. Other factors, including local anaesthetics (Villarejo and Case, 1984), pH (Heyde and Portalier, 1987; Sato *et al*., 2000) and nutrition limitation (Liu and Ferenci, 1998), also influence *ompF* and *ompC* transcription in an EnvZ/OmpR-dependent manner.

In recent years, biogenesis of OMPs has received renewed attention owing to the discovery of the β-barrel OMP assembly machine (Bam), comprised of four lipoproteins (BamBCDE) and a β-barrel OMP, BamA (Wu *et al*., 2005; Misra, 2007; Sklar *et al*., 2007). In the event of OMP misassembly or other envelope aberrations, cells invoke regulatory responses to minimize the envelope stress. Two principal regulators that control these responses are a specialized sigma factor σ^E^ (Alba and Gross, 2004) and the CpxA/CpxR two-component system (Raivio and Silhavy, 2001). An inner membrane protein, RseA, normally sequesters σ^E^ but misassembly of OMPs triggers two-step proteolysis of RseA, releasing σ^E^. Free σ^E^ activates transcription of factors involved in OMP biogenesis, including periplasmic foldases SurA, Skp; a periplasmic foldase/protease, DegP; members of the Bam complex; genes involved in lipopolysaccharide biogenesis; and small RNAs that inhibit OMP synthesis (Rhodius *et al*., 2006). Thus genes activated by the σ^E^ regulon help maintain a homoeostasis between OMP synthesis and OMP assembly. In contrast to the activation of σ^E^, a diverse group of envelope stresses appear to activate the CpxA/CpxR system (DiGiuseppe and Silhavy, 2003). Once activated, the CpxA/CpxR system induces the synthesis of periplasmic folding/degradation factors, including DsbA, PpiA and DegP, indicating that regardless of the source of envelope aberration, the ultimate targets of the CpxA/CpxR system include misfolded envelope proteins.

The robustness of the envelope homeostasis loop can be compromised if cells lack one or more OMP folding and/or degradation factors (Rizzitello *et al*., 2001; Onufryk *et al*., 2005). An *Escherichia coli* mutant simultaneously lacking DegP and BamB displays a conditional lethal (temperature-sensitive) phenotype, i.e. unable to grow at 37°C, but forms small but homogeneous colonies at 30°C (Charlson *et al*., 2006). The absence of BamB also causes antibiotic hypersensitivity to both hydrophobic (e.g. rifampin) and hydrophilic (e.g. vancomycin) antibiotics (Eggert *et al*., 2001; Vuong *et al*., 2008). In the present study, the temperature and antibiotic sensitivity phenotypes of Δ*bamB*Δ*degP* were exploited to isolate multicopy and mutational suppressors.

Isolation of multicopy suppressors identified a novel gene, *mzrA (yqjB*), while mutational suppressors identified pleiotropic *envZ* mutations. These mutations affect the expression of genes that are archetypal members of the EnvZ/OmpR regulon, such as *ompF* and *ompC*, and also influence the expression of genes, including *lamB* and *malE*, normally not controlled by the EnvZ/OmpR regulon (Wandersman *et al*., 1980; Case *et al*., 1986). Characterization of these suppressors showed that overexpression of *mzrA (yqjB*) modulates the EnvZ/OmpR two-component signal transduction regulon in a manner similar to the *envZ* suppressor mutations. The discovery of this property of *yqjB* led to its new designation, *mzrA*–*m*odulator of Env*Z* and Omp*R**A*. The phenotypic convergence of the two classes of suppressors suggests that they work through a similar mechanism to reduce envelope stress; in part, this entails reduction in OMP synthesis. Consistent with this view, Guillier and Gottesman (2006) previously showed that multicopy plasmid clones carrying *exuT*′*exuRyqjABC*′ activate *omrA* and *omrB* via EnvZ/OmpR.

## Results

### Overexpression of *mzrA (yqjB)* mitigates envelope stress

To identify potentially new genes involved in the response to envelope stress, we introduced a random plasmid gene library, prepared from the MC4100 chromosome, into the Δ*bamB*Δ*degP* strain and sought transformants that improved growth at 37°C. DNA sequence analysis of the insert DNA from four positive clones showed that three contained the intact *yfgM-bamB* genes while one clone carried a contiguous chromosomal region containing *exuT*′, *exuR*, *yqjA*, *mzrA (yqjB*), *yqjC*, *yqjD* and *yqjE′* genes. We focused on the *exuT-yqj* clone because the *yfgM-bamB* clone most likely improved growth by complementing the Δ*bamB* mutation of the recipient strain. Even though the *exuT-yqj* plasmid was isolated among clones that improved growth of the Δ*bamB*Δ*degP* strain at 37°C, transformants purified at 37°C formed small colonies. At 30°C, they formed colonies that were much larger than those formed by the parental Δ*bamB*Δ*degP* strain. Subsequent subcloning experiments narrowed the gene responsible for improved growth of the Δ*bamB*Δ*degP* strain to *mzrA (yqjB*).

**Fig. 1:**
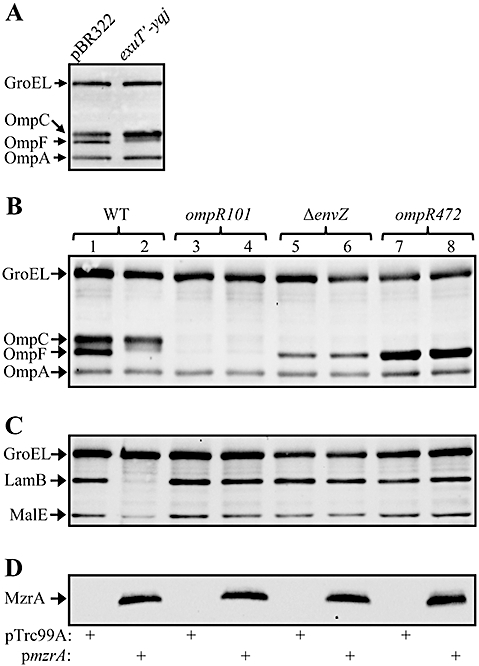
A. Effect of the *exuT-yqj* clone on OMPs in a Δ*bamB*Δ*degP* background. OMPs from cells grown overnight at 30°C were examined by Western blots using OmpF/OmpC/OmpA antibodies. B and C. Effect of MzrA (YqjB) overexpression on OmpA, OmpC and OmpF (B), and LamB and MalE (C) in different genetic backgrounds: *envZ*^+^/*ompR*^+^ (lanes 1 and 2), *envZ*^+^/*ompR101* (lanes 3 and 4), Δ*envZ*/*ompR*^+^ (lanes 5 and 6) and *envZ*^+^/*ompR472* (lanes 7 and 8). Indicated proteins were identified by Western blot analysis from freshly grown (37°C) cells using (B) GroEL and OmpF/OmpC/OmpA antibodies or (C) GroEL, LamB and MalE antibodies. GroEL serves as a gel loading control. Leaky (without the addition of IPTG) expression of *mzrA* from a pTrc99A-*mzrA* plasmid was sufficient to observe the effects of MzrA. D. Detection of MzrA_FLAG_ from cells analysed in (B) and (C) by FLAG antibodies. The presence of pTrc99A or pTrc99A-*mzrA* plasmid is indicated by the plus signs.

To gain insight into the mechanism by which overexpression of MzrA (YqjB) improves the growth of the Δ*bamB*Δ*degP* strain, we first examined the OMP profile. We have previously shown that reduced OMP levels partially suppress the temperature-sensitive phenotype of Δ*bamB*Δ*degP* (Charlson *et al*., 2006). The presence of the original *exuT-yqj* clone in the Δ*bamB*Δ*degP* strain showed a marked reduction of OmpF from cell envelopes (Fig. 1A). In the subsequent experiment, we used a plasmid clone expressing MzrA (YqjB) from an IPTG-inducible promoter. Overexpression of MzrA (YqjB) from an IPTG-inducible promoter dramatically reduced levels of OmpF and LamB, and to some extent MalE, but not OmpC and OmpA. The effects of MzrA (YqjB) were just as pronounced in a *bamB*^+^*degP*^+^ strain (Fig. 1B and C; lanes 1 and 2) as they were in a strain deleted for these genes (data not shown). Thus, mitigation of envelope stress was independent of other known pathways and was remarkably similar to a class of suppressor mutations identified through our antibiotic selection of Δ*bamB*Δ*degP* that localized to *envZ (*see below; Fig. 8). This result prompted us to examine the relationship between MzrA (YqjB) and the EnvZ/OmpR two-component regulatory system in influencing OMP and MalE levels.

### MzrA (YqjB) functions through EnvZ/OmpR

Overexpression of MzrA (YqjB) produces phenotypes similar to that conferred by pleiotropic *envZ* alleles (Wanner *et al*., 1979; Wandersman *et al*., 1980). Pleiotropic *envZ* alleles are dependent on OmpR (Slauch *et al*., 1988). Thus, we determined whether the effects of MzrA (YqjB) were also OmpR-dependent. For this experiment, we employed a non-polar null allele of *ompR*, *ompR101* (Sarma and Reeves, 1977). As expected, expression of both OmpF and OmpC was abolished in an *envZ*^+^*ompR101* background, but LamB and MalE were expressed normally (Fig. 1B and C). In contrast to the effect seen in an *envZ*^+^*ompR*^+^ background (Fig. 1C, lane 2), overexpression of MzrA (YqjB) in an *envZ*^+^*ompR101* background failed to reduce LamB and MalE levels (Fig. 1C, lane 4), indicating that OmpR is required for the effect of MzrA (YqjB). To further evaluate a requirement of OmpR for the negative effect of MzrA (YqjB) overexpression on OMPs and MalE, we employed another well-studied *ompR* allele, *ompR472* (Hall and Silhavy, 1981), which produces an OmpF^+^ OmpC^-^ phenotype without influencing LamB and MalE expression (Fig. 1B and C, lane 7). Again, we failed to see any negative effect of MzrA (YqjB) overexpression on OmpF, LamB or MalE (Fig. 1B and C, lane 8), thus emphasizing the need for wild type OmpR to observe the effects of MzrA (YqjB).

Next we tested the requirement for EnvZ in MzrA (YqjB)-mediated effects. In the absence of EnvZ, OmpC is not expressed while OmpF is expressed at diminished levels (Fig. 1B, lane 5). The low OmpF expression seen in an *envZ* null background is thought to be due to the phosphorylation of OmpR by acetyl phosphate (Hsing and Silhavy, 1997). Although expression of OmpC and OmpF is affected, LamB and MalE expression is not significantly altered in an *envZ* null background (Fig. 1C, lane 5). If MzrA (YqjB) directly influences OmpR, regardless of how OmpR gets phosphorylated, we may still observe the negative effects of MzrA (YqjB) on OmpF, LamB and MalE in an *envZ* null background. On the other hand, if MzrA (YqjB) modulates EnvZ activity, then in an *envZ* null background overexpression of MzrA (YqjB) will fail to reduce OmpF, LamB or MalE levels. The data in Fig. 1 showed that overexpression of MzrA (YqjB) in an *envZ* null background was unable to exert a negative effect on OmpF, LamB or MalE (compare lanes 5 and 6 in B and C). It is important to note that MzrA expression from the pTrc99A clone was unaffected in various mutant OmpR and EnvZ backgrounds (Fig. 1D), demonstrating that a lack of negative effect on OMPs or MalE was not the result of reduced MzrA expression. These results support our hypothesis that MzrA (YqjB) influences EnvZ activity that then transforms OmpR into a form responsible for the pleiotropic phenotype. Thus, YqjB was renamed MzrA, for modulator of EnvZ and OmpRA and will be henceforth referred to by this designation.

### MzrA influences steady-state expression of porins independently of the known modulators

**Fig. 2:**
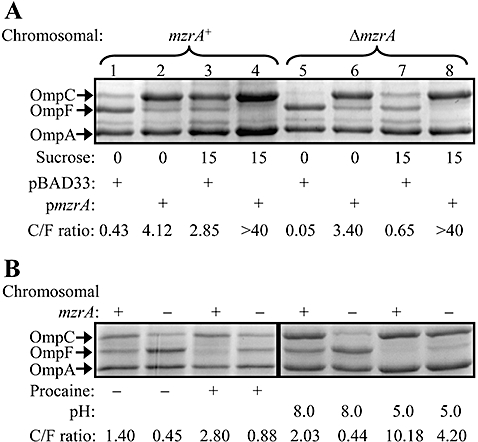
Effects of osmolarity, procaine and pH on porin expression in the presence or absence of MzrA. A. Envelopes from cells grown in medium A with (15%) or without (0) sucrose were analysed by SDS(urea)-PAGE and proteins were visualized by Coomassie blue stain. Strains used either had *mzrA* intact (lanes 1–4) or deleted (lanes 5–8) from the chromosome. Plus signs indicate presence of either a vector plasmid or an *mzrA* plasmid clone. All cultures were grown in the presence of 0.01% L-arabinose. B. For procaine's effect on porins, cells were grown in LB without salt and with (+) or without (−) 5 mM procaine. To assess the effect of pH on porins, cells were grown in 3-(N-morpholino)propanesulphonic acid (pH 8.0) or 2-(*N*-morpholino)ethanesulfonic acid (pH 5.0) buffered LB without salt. Envelopes from these cells were analysed by SDS(urea)-PAGE and protein bands were visualized after Coomassie staining. Plus and minus signs on top of the gel refer to the presence or absence, respectively, of the chromosomal *mzrA* gene. OmpC : OmpF ratios are shown.

As overexpression of MzrA affects porin expression in an EnvZ/OmpR-dependent manner, we investigated whether MzrA plays a role in the osmoregulation of porins. Envelopes were isolated from wild type and Δ*mzrA* strains carrying either an empty vector pBAD33 or pBAD33-*mzrA*_FLAG_. Cultures were grown in medium A (low osmolarity) and medium A supplemented with 15% sucrose (high osmolarity) (Kawaji *et al*., 1979). OMPs were analysed from envelopes by SDS (urea)-PAGE and protein bands were visualized by coomassie blue staining. In the wild type strain carrying just the empty vector, porin expression responded to medium osmolarity in an expected manner, i.e. high OmpF and low OmpC under low osmolarity and low OmpF and high OmpC under high osmolarity (Fig. 2A, lanes 1 and 3). In contrast, a significantly different porin expression profile was observed in the absence of MzrA, with OmpF being preferentially expressed regardless of the medium osmolarity (Fig. 2A, lanes 5 and 7). When MzrA was overexpressed, the porin expression profile switched in favour of OmpC, regardless of medium osmolarity (Fig. 2A, lanes 2, 4, 6 and 8). The fact that porin expression still responds to medium osmolarity in cells lacking or overexpressing MzrA suggets that MzrA is not the primarily receptor of medium osmolarity, rather, it appears to bias EnvZ/OmpR activity to favour OmpC expression over OmpF. In agreement with a recent report (Batchelor *et al*., 2005), we confirmed that deletion of *cpxR*, which only weakly reduces *mzrA* expression (see below), does not significantly influence porin expression or their osmoregulation (data not shown).

The local anaesthetic, procaine, is known to alter porin expression in an EnvZ/OmpR-dependent manner (Villarejo and Case, 1984) similar to pleiotropic *envZ* mutations (Villarejo and Case, 1984; Case *et al*., 1986) and MzrA overexpression (Fig. 1). Porin expression in a strain deleted for *mzrA* still responded to the presence of procaine in the same fashion as did the MzrA^+^ parental strain (Fig. 2B). Thus, MzrA does not mediate the effect of procaine on porin expression. Medium pH is also known to influence porin expression in an EnvZ/OmpR-dependent manner (Heyde and Portalier, 1987; Sato *et al*., 2000). At pH 8.0, OmpF to OmpC ratio in an *mzrA* strain was significantly higher than in the parental *mzrA*^+^ strain, but at pH 5.0 OmpC was the predominant porin in both strains (Fig. 2B), showing that pH-dependent porin regulation is independent of MzrA. Together, these data suggest that MzrA modulates the output activity of the EnvZ/OmpR system without influencing its ability to respond to various environmental signals.

### Evidence for MzrA's specificity

**Fig. 3:**
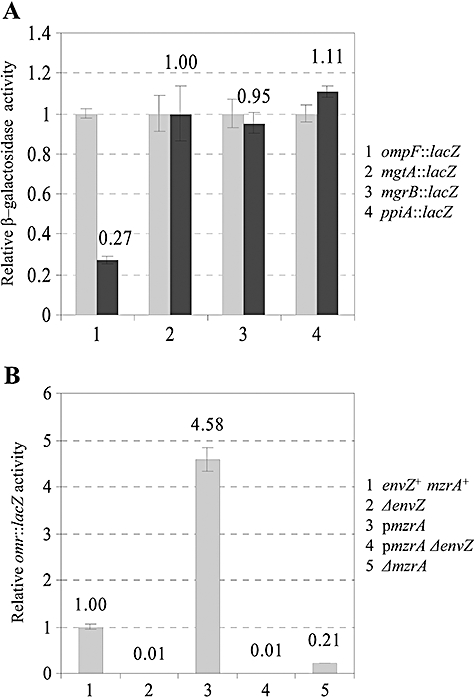
Specificity of MzrA. A. Effects of MzrA on *ompF*, *mgtA*, *mgrB* and *ppiA* expression were measured by assaying β-galactosidase activities of the chromosomal *lacZ* fusions to these genes. MzrA's expression from a plasmid was either not induced (light grey bars) or induced with 0.2% arabinose (dark grey bars). B. MzrA modulates the expression of *omr* genes through the EnvZ/OmpR system. Expression of *omr* was measured from a chromosomal *omr*::*lacZ* fusion. In some instances (B; 3 and 4), expression of MzrA from a pBAD24 clone was induced by the addition of 0.2% arabinose.

To test MzrA specificity for EnvZ and not other related membrane kinases, we used four different operon fusions whose activities are dependent on PhoP/PhoQ (*mgtA*::*lacZ*; *mgrB*::*lacZ*; Kato *et al.*, 1999), CpxA/CpxR (*ppiA*::*lacZ*; Pogliano *et al.*, 1997) or EnvZ/OmpR (*ompF*::*lacZ*; Hall and Silhavy, 1981). Overexpression of MzrA had no significant effect on *mgtA*::*lacZ*, *mgrB*::*lacZ* or *ppiA*::*lacZ* activities (Fig. 3A). We also used *cpxP*::*lacZ* as a second and perhaps a more sensitive target of the CpxA/CpxR regulon and found no appreciable change in its activity (a 1.13-fold increase) when MzrA was overexpressed. In contrast, overexpression of MzrA significantly reduced the activity of an EnvZ/OmpR-regulated *ompF*::*lacZ* fusion (Fig. 3A). Thus, the actions of MzrA appear to be specific to the EnvZ/OmpR regulon.

The *omrAB* genes were recently described as additional downstream targets of the EnvZ/OmpR regulon (Guillier and Gottesman, 2006). If MzrA activates the EnvZ/OmpR regulatory system then *omrAB* expression is expected to increase when MzrA is overproduced and decrease in the absence of MzrA. Consistent with this, *omr*::*lacZ* activity increased 4.5-fold in response to MzrA overexpression and this increase was absolutely dependent on the EnvZ/OmpR system (Fig. 3B). Moreover, in a strain deleted for *mzrA*, *omr*::*lacZ* activity decreased fivefold (Fig. 3B), showing that chromosomal expression of MzrA is sufficient to modulate the EnvZ/OmpR regulon.

Together, these results suggest that overexpression of MzrA activates the EnvZ/OmpR regulatory system, presumably by directly modulating EnvZ enzymatic activity. The activated EnvZ/OmpR system then influences the expression of a number of downstream target genes, such as *ompF*, *ompC* and *omrAB*, archetypal members of the EnvZ/OmpR regulon. Other affected genes include *lamB* and *malE*, which are normally not influenced by EnvZ/OmpR unless MzrA is overexpressed or in the presence of pleiotropic *envZ* mutations (Wanner *et al*., 1979; Wandersman *et al*., 1980).

### MzrA links CpxA/CpxR and EnvZ/OmpR regulons

**Fig. 4:**
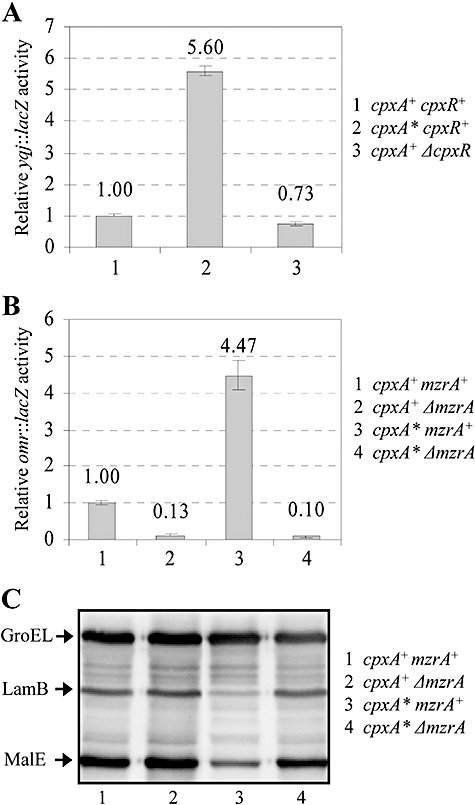
Expression of *omr*, *yqjAmzrA*, *lamB* and *malE* in various genetic backgrounds. Expression of *omr* (A) and *yqjAmzrA* (B) was measured by assaying the activities of the respective chromosomal *lacZ* fusions. LamB and MalE levels were measured by Western blot analysis (C) as described in the Fig. 1 legend.

The *yqjA* and *mzrA* open reading frames are separated by three nucleotides and are thought to be cotranscribed from σ^70^(p1)- and σ^E^(p2)-dependent promoters located upstream of *yqjA* (Dartigalongue *et al*., 2001). Moreover, the *yqjAmzrA* operon has been shown to be regulated by the CpxA/CpxR two-component regulatory system (Yamamoto and Ishihama, 2006), which responds to envelope stress (Raivio and Silhavy, 2001). To corroborate the previous results, we measured the β-galactosidase activity of a chromosomal *yqj*::*lacZ* fusion. In a constitutively activated CpxA* mutant background (Cosma *et al*., 1995) the activity increased 5.6-fold (Fig. 4A). Interestingly, in the absence of CpxR, *yqj*::*lacZ* activity decreased only modestly (27%), indicating that additional regulator(s) may play a role in activating *yqjAmzrA*. As mentioned above, *yqjAmzrA* was reported to be regulated by a specialized sigma factor, σ^E^. We found that *yqj*::*lacZ* activity was slightly increased (1.5-fold) in a Δ*rseA* background in which σ^E^ is fully active (Alba and Gross, 2004).

As MzrA functions through EnvZ/OmpR (Fig. 1), it stands to reason that the activated CpxA/CpxR system may influence the EnvZ/OmpR regulon via MzrA. To test this possibility, we used an *omr*::*lacZ* chromosomal fusion whose expression is dependent on EnvZ/OmpR (Fig. 4B). In a constitutively activated CpxA*/CpxR background, the *omr*::*lacZ* activity rose more than fourfold over that present in a CpxA^+^ CpxR^+^ background, and this increase was dependent on MzrA (Fig. 4B). Furthermore, it can be seen in Fig. 4C that activated CpxA*/CpxR downregulates LamB and MalE expression in an MzrA-dependent manner, thus providing further evidence for an MzrA-mediated link between these two-component systems. The absence of MzrA did not alter the effect of CpxR on *ompF* and *ompC (*data not shown). This is not surprising because CpxR has been shown to directly influence *ompF* and *ompC* (Batchelor *et al*., 2005).

### MzrA is an inner membrane protein

**Fig. 5:**
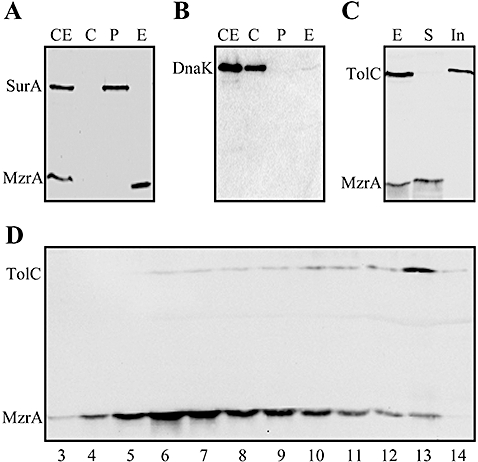
Localization of MzrA. Freshly grown cultures expressing MzrA_FLAG_ from pBAD24 plasmid were fractionated into various cellular compartments and proteins from various fractions were visualized through Western blot analysis. Proteins samples from whole-cell extract (CE), cytoplasm (C), periplasm (P) and envelopes (E) fractions were probed with FLAG- and SurA- (A) or DnaK (B) specific antibodies. C. Whole-cell envelopes (E) were treated with 0.3% sarcosyl and proteins from sarcosyl soluble (S) and sarcosyl insoluble pellet (In) were analysed by a Western blot using TolC- and FLAG-specific antibodies. D. Sucrose density gradient analysis of cell envelopes (E) obtained from a strain expressing MzrA_FLAG_. Protein samples from fractions 3–14 were analysed by Western blot analysis using TolC- and FLAG-specific antibodies. MzrA_FLAG_ peaks with the inner membrane fractions 6 and 7, while TolC peaks with an outer membrane fraction 13.

MzrA is a 127-residue protein with a theoretical p*I* of 9.51. It contains a single predicted *trans*-membrane (TM) domain extending from residues 11 to 31 of the polypeptide chain (Fig. S1). To determine the cellular localization of MzrA, we constructed a fully functional variant of MzrA containing a C-terminal FLAG tag. Fractionation of cells into the cytoplasm, the periplasm and the envelopes showed that MzrA is an envelope protein (Fig. 5A). SurA and DnaK were present only in the periplasmic and cytoplasmic fractions respectively (Fig. 5A and B), thus confirming the purity of various fractions.

We then examined whether MzrA localizes to the inner or outer membrane by employing two methods. In the first method, which involves differential solubilization of membrane proteins, we found that a 0.3% solution of sarcosyl fully solubilized MzrA_FLAG_ from the envelopes, whereas TolC, an OMP, remained insoluble (Fig. 5C). This indicated that MzrA is an inner membrane protein. The location of MzrA was confirmed by sucrose density gradient analysis (Fig. 5D), which showed that MzrA localizes with the lighter density inner membrane fractions (buoyant density of around 1.15 g cm^−3^). In contrast, TolC fractionated with the heavier density outer membrane fractions (buoyant density of around 1.22 g cm^−3^). As a control marker for the inner membrane proteins, we excised and sequenced a protein band whose presence peaked in the same fractions as MzrA and determined it to be AtpD, the inner membrane-localized β-subunit of ATPase. Together, these analyses established that MzrA is an inner membrane protein.

### The C-terminus of MzrA is exposed to the periplasm

**Fig. 6:**
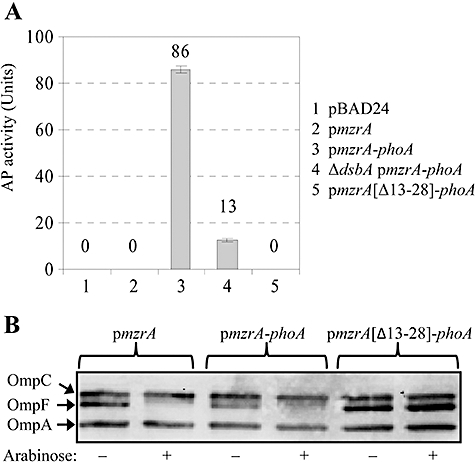
Bi-functional nature of MzrA::PhoA. A. Alkaline phosphatase (AP) activities of cells expressing various plasmid clones in the presence (lanes 1–3 and 5) or the absence (lane 4) of the periplasmic disulphide bond isomerase DsbA. B. Functionality of MzrA of MzrA::PhoA, with or without the putative TM domain of MzrA, was assessed by analysing OmpF levels. Cells carrying various plasmids constructs, as shown on top of the gel, were grown without (−) or with (+) arabinose (0.2%) to induce the expression of plasmid-borne MzrA or fusion proteins. Protein extracts from these cells were analysed by Western blots using antibodies to detect OmpF, OmpC and OmpA. Expression of plasmid-borne proteins was induced with 0.2% arabinose.

The presence of a single putative N-terminal TM domain renders the possibility that the C-terminal domain of MzrA is either exposed in the cytoplasm or in the periplasm. To determine its topology, we first constructed a C-terminal MzrA-reporter gene fusion with PhoA in which codons corresponding to the mature PhoA protein were fused downstream of the MzrA open reading frame. The alkaline phosphatase activity of PhoA is functional in the oxidizing environment of the periplasm (Boyd *et al*., 1987). The resulting MzrA::PhoA fusion was bi-functional, indicating that its expression not only stimulated alkaline phosphatase activity (an 80-fold increase in alkaline phosphatase activity over background chromosomal activity; Fig. 6A), but also conferred MzrA-specific phenotypes, such as inhibition of OmpF (Fig. 6B). MzrA::PhoA activity in a *dsbA* background, deficient in periplasmic oxidoreductase activity, was reduced over eightfold compared with a *dsbA*^+^ background (Fig. 6A), confirming the periplasmic orientation of MzrA::PhoA. An in-frame deletion removing residues 13–28 of the putative TM domain of MzrA from the MzrA::PhoA fusion completely abolished its alkaline phosphatase activity and failed to reduce OmpF level (Fig. 6B), indicating a requirement of the TM domain for membrane localization and activity. We also constructed MzrA::αLacZ fusions and used them in conjunction with the LacZ ω fragment, to test the orientation of the C-terminus of MzrA. The Lac complementation data obtained from these experiments corroborated the conclusion drawn from the MzrA::PhoA data (data not shown). These results provided strong genetic evidence that the MzrA C-terminus is exposed in the periplasm. Evidence that the N-terminus of MzrA is exposed in the cytoplasm came from Cya fusion studies described below.

### MzrA interacts with EnvZ

**Fig. 7:**
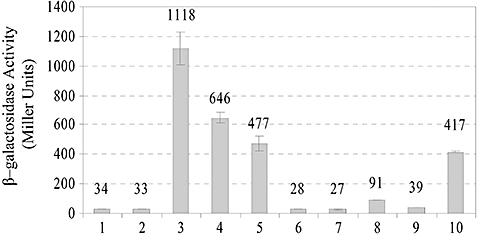
Interactions between MzrA and EnvZ. A bacterial two-hybrid system was used to detect *in vivo* protein–protein interactions. Values of β-galactosidase activities, calculated in Miller Units, with standard errors are shown: (1) pT25/pT18, (2) pT25::MzrA/pT18, (3) pT25::MzrA/pT18::MzrA, (4) pT25::MzrA/pEnvZ::T18, (5) pT25::MzrA/pT18::EnvZ, (6) pT25::MzrA/pT18::OmpR, (7) pT25::MzrA/pOmpR::T18, (8) pT25::MzrA/pCpxA::T18, (9) pT25::MzrA/pT18::CpxA and (10) pT25::EnvZ/pEnvZ::T18.

The genetic data presented thus far suggested that MzrA modulates the EnvZ/OmpR regulon by influencing EnvZ (Fig. 1). To determine if MzrA interacts with EnvZ *in vivo*, we employed a bacterial two-hybrid reporter system based on functional complementation between *Bordetella pertussis* adenylate cyclase fragments, T18 and T25, expressed separately from two compatible plasmid replicons (Karimova *et al*., 1998). Adenylate cyclase activity is restored only when proteins fused to T18 and T25 interact. Functional reconstitution of *B. pertussis* adenylate cyclase in an *E*. *coli*Δ*cya lac*^+^ strain is monitored by assaying the activity of a cAMP-CRP-dependent *lac* promoter of a chromosomally encoded *lac* operon. Thus, measurement of the β-galactosidase activity provides a convenient and sensitive means of detecting *in vivo* protein–protein interactions. Fusions were constructed with T18 and T25 present at the N- or C-terminal of EnvZ (both termini of EnvZ are exposed to the cytoplasm). Only N-terminal MzrA T18/T25 fusions were constructed because the topology data above indicated that the C-terminus of MzrA is exposed in the periplasm and therefore, C-terminal T18 and T25 fusions are not expected to produce functional complementation. We also constructed OmpR fusions to detect their interactions with MzrA. Lastly, to test the specificity of this *in vivo* assay, we made N- and C-terminal T18 fusions with the membrane kinase protein, CpxA. All plasmids expressing fusion constructs were transformed into a Δ*cya lac*^+^ strain and functional complementation was assessed by measuring β-galactosidase activity of the chromosomally encoded *lacZ* gene (Fig. 7). Formation of MzrA and EnvZ homo-dimers or homo-oligomers was evident by a 33- and 12-fold (lanes 3 and 10) increase in β-galactosidase activity over the control strain (lane 2).

Importantly, formation of MzrA-EnvZ heteromers was readily apparent from two different EnvZ constructs resulting in a 19- and 14-fold increase in β-galactosidase activity (lanes 4 and 5). In contrast, no significant MzrA–OmpR interactions (lanes 6 and 7), or MzrA–CpxA interactions (lanes 8 and 9) were detected. Thus, MzrA interacts with EnvZ, but not with OmpR or CpxA.

### Pleiotropic *envZ* mutations suppress *ΔbamB ΔdegP*

Concurrent with the multicopy suppressor analysis, we carried out a genetic analysis to identify mutational suppressors of Δ*bamB*Δ*degP*. These suppressors were isolated at 30°C and 37°C on Luria broth (LB) agar (LBA) medium supplemented with vancomycin. Subsequent characterization showed that, as expected, they displayed reduced sensitivity towards vancomycin, but also to other antibiotics, such as rifampin, to which the parental strain is also sensitive. Curiously, even though some suppressors of this class were isolated at 37°C, they failed to form regular-sized colonies at 37°C; however, at 30°C all suppressors formed large homogeneous colonies similar to the *bamB*^+^*degP*^+^ strain.

**Fig. 8:**
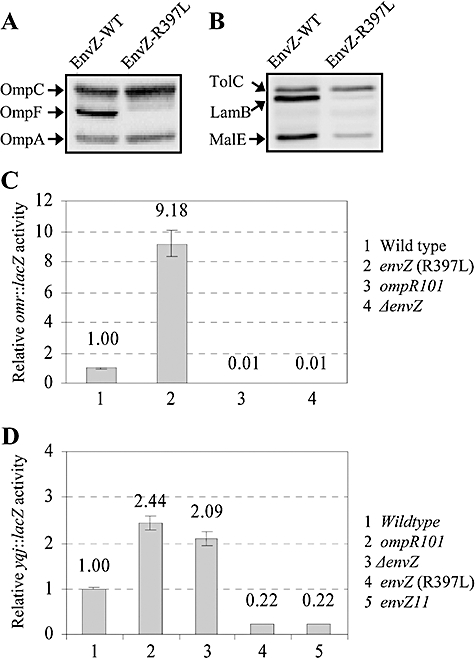
Effect of an *envZ* suppressor allele on OMPs, MalE and *omr*. Protein extracts expressing wild type (WT) or mutant (EnvZ-R397L) EnvZ were analysed by Western blots using (A) OmpF/OmpC/OmpA antibodies or (B) TolC, LamB and MalE antibodies. OmpA and TolC serve as gel loading controls. *omr* (C) or *yqjAmzrA* (D) expression were assessed by measuring the activity of the respective chromosomal *lacZ* fusions, in genetic backgrounds shown in the figures.

Examination of OMPs and MalE by Western blot analysis from revertants showed that they had significantly reduced levels of OmpF, LamB and MalE, but the levels of OmpC and OmpA were not reduced. These characteristics persisted even when the Δ*bamB*Δ*degP* alleles were replaced by the corresponding wild type alleles (Fig. 8A and B). To assess whether the reduction in OmpF levels was due to an effect on *ompF* transcription, we introduced a chromosomal *ompF*::*lacZ* operon fusion via a linked Tn*10* (Tc^r^) marker into a strain carrying the suppressor mutation. In the suppressor strain, *ompF*::*lacZ* activity was found to be extremely low (light blue colonies on Xgal plates) compared with that of the parental strain (dark blue colonies on Xgal plates). We exploited this *ompF*::*lacZ*-down phenotype of the suppressor mutation to isolate plasmid clones carrying a wild type copy of the suppressor gene. DNA sequence analysis of plasmid clones partially reversing the Lac-down phenotype revealed that they contained a contiguous chromosomal region with intact *greB*, *ompR* and *envZ* genes. Direct DNA sequencing of this region of the chromosome from suppressor isolates revealed that they carried *envZ* mutations, resulting in a W134C, F284C or R397L substitution or a five-amino-acid insertion after residue 26 of EnvZ. Introduction of one of the mutant *envZ* alleles, causing a R397L substitution, in a strain expressing a chromosomal *omr*::*lacZ* fusion resulted in an over ninefold increase in the β-galactosidase activity (Fig. 8C), showing the constitutively activated nature of this *envZ* allele (and presumably the other three *envZ* alleles as well). Based on OMP and MalE profiles, this class of *envZ* suppressor mutations resembles the previously described pleiotropic *envZ* alleles (Wanner *et al*., 1979; Wandersman *et al*., 1980). Therefore, we also tested the previously characterized pleiotropic *envZ11* and *envZ473* alleles and found that they too suppressed the temperature and drug sensitivity phenotypes of the Δ*bamB*Δ*degP* strain (data not shown).

### *mzrA* expression is negatively controlled by EnvZ/OmpR

Kato *et al*. (2003) discovered a negative regulatory feedback loop in which the expression of *pmrD* was shown to be negatively regulated by PmrA, the response regulator of the PmrB/PmrA two-component system; PmrD has been shown to promote phosphorylation of PmrA (Kato and Groisman, 2004). We asked whether such feedback-negative regulatory loop also exists in case of *mzrA*-EnvZ/OmpR. In the absence of EnvZ or OmpR, the expression of *yqj*::*lacZ* went up twofold (Fig. 8D). Moreover, in constitutively activated EnvZ mutant backgrounds expressing either EnvZ11 or EnvZ (R397L), the expression of *yqj*::*lacZ* was reduced almost fivefold (Fig. 8D). These results are consistent with a notion that the expression of *mzrA*, whose product modulates EnvZ/OmpR, is feedback-inhibited by these regulators.

### The mechanistic basis of the pleiotropic *envZ* phenotype

In order to understand the underlying effect of the EnvZ substitution on its activity, we overexpressed and purified the cytoplasmic domain of wild type EnvZ (EnvZc) and one of the EnvZ mutants (R397L) for which we have already presented *in vivo* data in Fig. 8. We compared their autophosphorylation, phosphotransfer to OmpR and dephosphorylation properties *in vitro*.

**Fig. 9:**
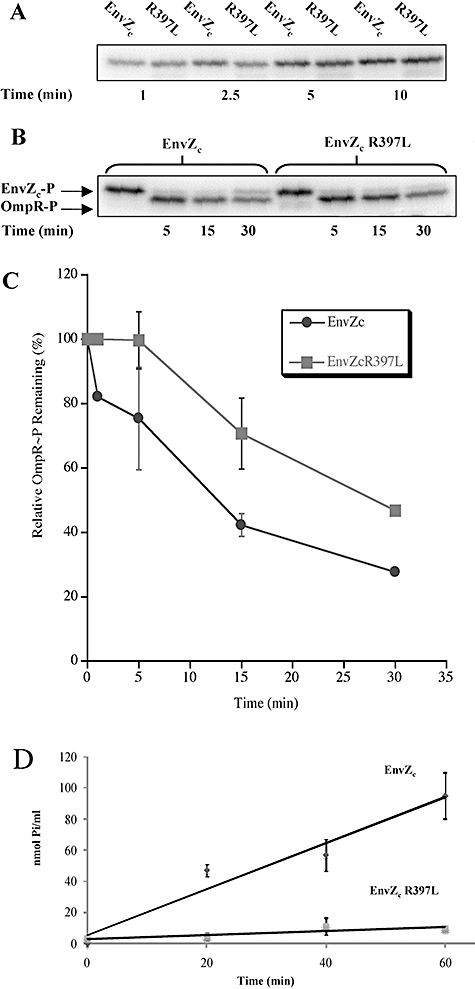
A. EnvZ_c_ and EnvZ_c_ R397L display similar rates of autophosphorylation. EnvZ_c_ (and EnvZ_c_ R397L) were added at a final concentration of 4 μM. Time of incubation is as follows: lanes 1 and 2 (1 min), lanes 3 and 4 (2.5 min), lanes 5 and 6 (5 min) and lanes 7 and 8 (10 min). Each reaction was stopped with 3 μl of denaturing sample buffer and 10 μl of the 20 μl reaction was loaded on the gel. B. OmpR∼P persists after phosphorylation by EnvZ_c_ R397L. EnvZ_c_ or EnvZ_c_ R397L was added at a final concentration of 4 μM. OmpR was added at a final concentration of 2 μM. Lanes 1 and 5 contain the results of a 10 min autophosphorylation reaction with EnvZ_c_ or EnvZ_c_ R397L respectively. Phosphotransfer to OmpR is shown in lanes 2–4 and 6–8 with the time of each reaction indicated below each lane. Reactions were stopped by the addition of 3 μl of denaturing sample buffer, and 10 μl of the 20 μl reaction was loaded on the gel. C. Densitometric analysis. Densitometry on the experiment shown in (B) (and a repeat experiment) was performed using Molecular Dynamics ImageQuant Software, version 5.0. The results are plotted as a per cent of the EnvZ∼P determined as a function of time, the symbol represents the mean and the error bars indicate the range of two separate experiments. The absence of error bars at 1 and 30 min was due to the elimination of that time point in the second experiment. D. The EnvZ_c_ R397L ATPase is reduced by 10-fold compared with the wild type EnvZ_c_. ATPase assays were performed with EnvZ_c_ (2 μM) and the EnvZ_c_ R397L mutant (2 μM) in the presence of OmpR (1 μM). Reactions were performed in triplicate and the standard deviation at each time point is indicated in parentheses. For EnvZ_c_, the values at 20, 40 and 60 min are 47.01 (3.86), 56.90 (9.95) and 95.08 (14.85) respectively. For EnvZ_c_ R397L, the values at the same time points are 4.42 (2.26), 11.09 (5.51) and 9.10 (0.59) respectively. The ATPase activity was expressed as nanomoles of P_i_ liberated per ml (see *Experimental procedures* for details).

We first examined EnvZ autophosphorylation and phosphotransfer to OmpR by incubating EnvZc and EnvZ R397Lc with [γ-^32^P]-ATP for various times and analysing the products via autoradiography after separation on sodium dodecyl sulphate-polyacrylamide gel electrophoresis (SDS-PAGE) (Fig. 9A). The wild type and mutant activities are directly compared over 10 min time-course. It is apparent that the wild type and the mutant have quite similar autophosphorylation levels. We selected 10 min time point for EnvZ autophosphorylation to compare the phosphotransfer to OmpR (Fig. 9B). After addition of OmpR, the phosphoryl group from EnvZ∼P is transferred to OmpR and the mass of the labelled band shifts (lanes 2–4 and 6–8). By visual inspection, the phosphotransfer activities of the wild type and the mutant appear somewhat similar. However, densitometric analysis of OmpR∼P (produced by EnvZ∼P or EnvZcR397L∼P) indicated that at every time point, there was more OmpR∼P present when phosphorylated by EnvZcR397L compared with the wild type (Fig. 9C). This is most dramatic at 15 min, where there was nearly 50% more OmpR∼P present. We then measured EnvZ ATPase activity. EnvZ, phosphorylated directly from ATP, transfers its histidyl phosphate to OmpR and subsequently stimulates OmpR∼P dephosphorylation. This results in the net breakdown of ATP and the subsequent release of inorganic phosphate from OmpR∼P. The sum of these reactions can therefore be analysed by measuring ATP hydrolysis (Kenney, 1997; Mattison and Kenney, 2002). We measured inorganic phosphate (P_i_) production over time at a ratio of 2:1 EnvZ to OmpR (Fig. 9D). It is apparent from the figure that the EnvZ R397L mutant has an approximately 10-fold lower turnover rate (0.12 μmol Pi mg^−1^ h^−1^) compared with the wild type (1.48 μmol Pi mg^−1^ h^−1^). As we have already shown that the EnvZ mutant is not defective in its autophosphorylation (Fig. 9A) and early phosphotransfer appears to be similar (data not show) (Fig. 9B and C), a dramatic reduction in the turnover observed in the presence of the EnvZ R397L mutant in the ATPase assay (Fig. 9D) may be due to the diminished ability of the mutant EnvZ to dephosphorylate OmpR∼P, thus increasing its half-life. It is worth noting that previously characterized pleiotropic EnvZ mutants were also shown to have diminished phosphatase activity, thus resulting in an elevated half-life of OmpR∼P (Aiba *et al*., 1989; Tokishita *et al*., 1991; Hsing *et al*., 1998). Presumably *in vivo*, this increased stability promotes OmpR binding at promoters that it does not normally regulate, resulting in a pleiotropic phenotype. Alternatively, constitutive activation of normally OmpR-regulated promoters ultimately leads to secondary regulatory cascades that are responsible for the phenotypes observed.

## Discussion

Characterization of suppressors that overcome envelope stress caused by Δ*bamB*Δ*degP* led to the discovery of a novel protein, MzrA, and pleiotropic mutations in *envZ*. Their suppressive effects are possibly due in part to reduced synthesis of two OMPs, OmpF and LamB, thereby reducing the OMP load on compromised assembly machinery. As both suppressors also lower the Δ*bamB* drug sensitivity phenotype, which cannot be fixed by deleting the *ompF* and *lamB* genes, we suspect additional alterations in the membrane structure are responsible for mending the outer membrane permeability breach of the Δ*bamB*Δ*degP* strain.

The EnvZ/OmpR two-component regulatory system has long been studied for its involvement in the osmoregulation of porins. Although an exhaustive amount of research has been carried out in this area, it remains unclear as to how EnvZ senses environmental stimuli and how this sensing alters its autokinase, phosphotransferase and phosphatase activities. Results presented in this study showed that MzrA, a small inner membrane protein, is an upstream regulator of the EnvZ/OmpR system. Although the porin expression profile in cells either lacking or overexpressing MzrA was drastically different compared with that seen in wild type MzrA^+^ cells, porin expression still responded to changes in medium osmolarity, pH or the presence of procaine in the medium, suggesting that MzrA is not the primary receptor of these three environmental cues that influence porin expression via EnvZ/OmpR. Based on the porin expression profile when MzrA is overexpressed (high OmpC, low OmpF regardless of the medium osmolarity), it appears that MzrA modulates the EnvZ/OmpR system to favour the accumulation of OmpR∼P (Fig. 9). This could be achieved by an elevated autokinase activity, diminished phosphatase activity or a combination of the two. Because OmpR has a high non-specific binding component and lacks highly conserved binding sites, it is a good global regulator (Rhee *et al*., 2008). Increasing the half-life of OmpR∼P (through *envZ* mutations or MzrA overexpression) would facilitate higher-affinity interaction with DNA (Head *et al*., 1998) and would enable OmpR to bind to promoters it does not normally regulate.

Using a bacterial two-hybrid system, we showed that MzrA interacts with EnvZ. Exactly how the binding of MzrA to EnvZ modulates EnvZ enzymatic activity remains to be determined, but we demonstrated that it does so by increasing OmpR∼P stability. MzrA binding might promote a conformation of EnvZ that exists in the presence of procaine or when EnvZ is constitutively altered by pleiotropic *envZ* mutations found here and isolated previously (Wanner *et al*., 1979; Wandersman *et al*., 1980). Although at present there is no biochemical evidence showing MzrA increases OmpR∼P stability, the observation that overexpression of MzrA produces a phenotype that is almost identical to that seen in a strain expressing a pleiotropic *envZ* allele, indicates that the biochemical state of EnvZ/OmpR in these two genetic backgrounds is also very similar; i.e. both backgrounds likely increase OmpR∼P stability.

The MzrA TM region is not very conserved among its homologues, which are found in at least nine members of the *Enterobacteriaceae* family (Fig. S1). The role of the TM domain may be to target the protein to the inner membrane and this was evident from the MzrA::PhoA data. In contrast, the central region of MzrA, which is exposed to the periplasm, contains conserved residues that may make direct contact with the periplasmic domain of EnvZ. We suggest that residues of the MzrA periplasmic domain play an important role in modulating EnvZ enzymatic activity. An earlier search for a periplasmic protein ligand that interacts with the periplasmic domain of EnvZ was unsuccessful (Egger and Inouye, 1996). Our finding that MzrA is an inner membrane protein would explain why MzrA was not identified in the previous biochemical screen where only soluble periplasmic proteins were probed.

**Fig. 10:**
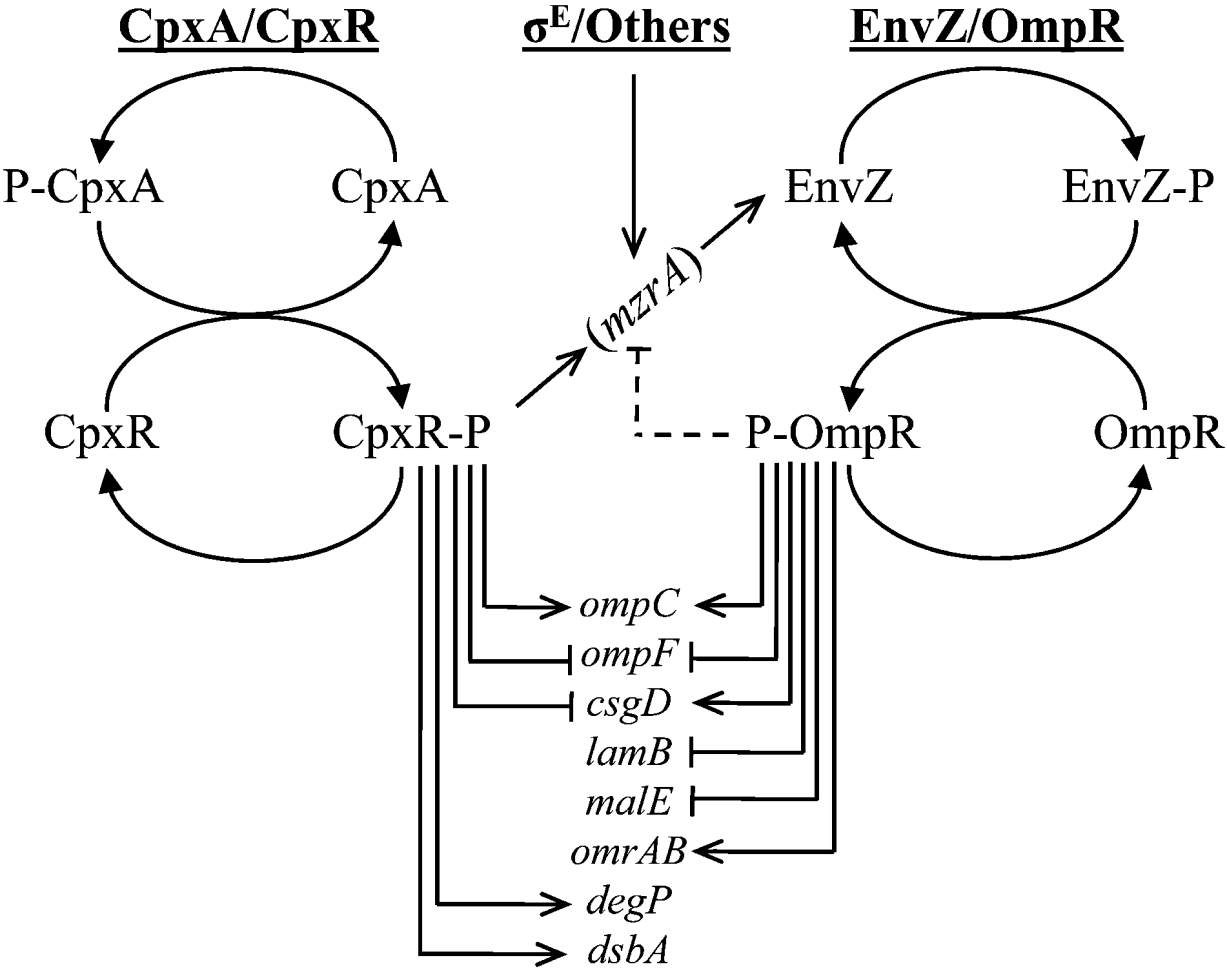
MzrA-mediated bridging of the CpxA/CpxR and EnvZ/OmpR regulons. The activated CpxA/CpxR and σ^E^ regulons turn on the synthesis of MzrA, which, in turn through its interaction with EnvZ, modulates EnvZ/OmpR's activity to favour the accumulation of OmpR∼P. High levels of OmpR∼P upregulates the expression of *ompC*, *csgD* and *omrAB*, but downregulates the expression of *ompF*, *lamB* and *malE*. High OmpR∼P also downregulates *mzrA*, thus establishing a negative-regulatory feedback loop. Also shown are genes that are independently or uniquely affected by the CpxA/CpxR regulon. Not all genes that may be affected by the activated CpxA/CpxR and EnvZ/OmpR regulons are shown. Others refer to unknown factors that may regulate MzrA's synthesis.

We have provided evidence that MzrA links two major signal transduction regulatory systems: CpxA/CpxR and EnvZ/OmpR, which respond to different environmental and cellular signals to regulate outer membrane composition. The network of genes regulated by the CpxA/CpxR and EnvZ/OmpR systems is not entirely unique; for example, both systems independently regulate transcription of *csgD*, *ompC* and *ompF* (Batchelor *et al*., 2005; Jubelin *et al*., 2005). However, the environmental cues to which they respond are distinct; whereas alkaline pH or misfolded pilus subunits activate CpxA/CpxR, medium osmolarity is the best-characterized effector of EnvZ/OmpR. Thus, despite responding to different stimuli, MzrA-mediated linking of the CpxA/CpxR and EnvZ/OmpR regulatory systems would allow cells to amplify their response to appropriately adjust the envelope composition (see Fig. 10).

Bridging of separate two-component regulatory systems by a membrane protein is not unique to MzrA. Recently, a small protein, B1500, of *E*. *coli* has been shown to connect the EvgS/EvgA and PhoQ/PhoP two-component signal transduction pathways (Eguchi *et al*., 2007). B1500 is a relatively weakly conserved protein, as it is only found in *E*. *coli* and highly related species of *Shigella*. In contrast, MzrA, which is unrelated to YqjB from *Bacillus*, is a relatively well-conserved protein of the *Enterobacteriaceae* family (Fig. S1), thus signifying its widespread physiological role. Although there is no sequence homology between MzrA and B1500, and MzrA is almost twice as large as B1500, their membrane topology and possible mode of action on cognate sensor kinases appear to be very similar.

Modulators of bacterial two-component systems are not limited to inner membrane-localized proteins, such as MzrA and B1500. For example, two soluble proteins, CpxP and PmrD, have also been shown to modulate the CpxA/CpxR (Isaac *et al*., 2005) and PmrB/PmrA regulons (Kato and Groisman, 2004) respectively. CpxP is a periplasmic protein that is proposed to interact with the periplasmic domain of the sensor kinase CpxA to downregulate CpxA autokinase activity (Fleischer *et al*., 2007). On the other hand, cytosolic PmrD was shown to bind to the phosphorylated form of the response regulator, PmrA and stabilize PmrA∼P (Kato and Groisman, 2004). Interestingly, we discovered here that like the case with *pmrD*-PmrB/PmrA, *mzrA* expression is negatively regulated by EnvZ/OmpR that are post-translationally modulated by MzrA. Such negative feedback-regulatory loop appears to be well suited to re-establish homeostasis when the ‘signals’ that activate the *mzrA*-EnvZ/OmpR cascade have dissipated (Fig. 10).

The discovery of MzrA as a communicator between CpxA/CpxR and EnvZ/OmpR gives physiological credence to earlier claims of ‘cross-talk’ among different sensor kinases/response regulators (Laub and Goulian, 2007). Despite the existence of various cross-regulatory strategies, it is reasonable to consider that the genes affected by the primary regulon are the principal responders of a given stimulus and the genes influenced through cross-communicators are response amplifiers. For example, under envelope stress conditions, products of the genes including *degP*, *ppiA* and *dsbA* that are regulated by CpxA/CpxR would directly respond to help reduce envelope stress (Raivio and Silhavy, 2001). However, the MzrA-mediated connection of CpxA/CpxR to EnvZ/OmpR would allow participation of *omrAB* to decrease envelope stress by reducing the synthesis of certain OMPs (Lundrigan and Earhart, 1981; Guillier and Gottesman, 2006), thus helping to maintain homeostasis between OMP assembly and synthesis.

Both the pleiotropic *envZ* mutations and overexpression of MzrA reduce the drug hypersensitivity phenotype of Δ*bamB*, against which they were isolated, but also of unrelated Δ*surA* and Δ*tolA* mutations. The root cause of the hypersensitivity phenotype conferred by the Δ*bamB*, Δ*surA* and Δ*tolA* mutations is presently unknown, but it is likely due to a compromised outer membrane permeability barrier. Formation of phospholipid bilayer patches in the outer membrane is often thought to be associated with sensitivity to hydrophobic antibiotics, such as rifampin (Nikaido and Vaara, 1985), but sensitivity to a hydrophilic antibiotic, vancomycin, may be due to the formation of ‘cracks’ through which this large antibiotic can slip through (Ruiz *et al*., 2006). The ability to reduce sensitivity to both classes of antibiotics suggests that pleiotropic *envZ* mutations and the overexpression of MzrA employ a common mechanism to repair the outer membrane permeability breach. Interestingly, *yqjA*, the first gene of the *yqjA-mzrA* operon, has been shown to be involved in some aspect of membrane permeability (Shi *et al*., 2004) and phospholipid synthesis (Thompkins *et al*., 2008). We found that MzrA influences porin expression via EnvZ/OmpR but independent of YqjA (data not shown). Similarly, the ability of MzrA to reduce drug hypersensitivity of a Δ*bamB* strain was found to be independent of YqjA. Thus, a functional relationship, if any, between YqjA and MzrA remains unknown.

## Experimental procedures

### Bacterial strains and chemicals

*Escherichia coli* K-12 strains used here were mostly derived from MC4100 (Casadaban, 1976) and are listed in the Table S1. The bacterial adenylate cyclase-based two-hybrid system strains and plasmids were purchased from Euromedex. JM109 (Promega) was used for LacZα complementation analysis. Immun-star HRP substrate was purchased from Bio-Rad. Enzyme-catalysed fluorescence substrate was purchased from GE Healthcare. Rabbit anti-FLAG polyclonal antibodies and goat anti-mouse IgG secondary antibodies were purchased from Sigma-Aldrich. Mouse anti-DnaK and goat anti-GroEL polyclonal antibodies were purchased from Stressgen. ONPG (2-ortho-Nitrophenyl-β-D-galactopyranoside) was from ACROS and ρ-NPP (ρ-Nitrophenyl Phosphate) was from Thermo Fisher Scientific. Procaine was purchased from MP Biomedicals. All other chemicals were of analytical grade.

The LB and LBA were prepared as described previously (Silhavy *et al*., 1984) and when required, supplemented with ampicillin (50 μg ml^−1^), chloramphenicol (12.5 μg ml^−1^), kanamycin (25 μg ml^−1^) or arabinose (0.01% or 0.2%). Low-osmolarity medium-A contains nutrient broth (7.0 g l^−1^), yeast extract (1.0 g l^−1^), K_2_HPO_4_ (3.7 g l^−1^), KH_2_PO_4_ (1.3 g l^−1^) and glycerol (2.0 g l^−1^) (Kawaji *et al*., 1979) and was supplemented with 15% sucrose for high osmolarity. For analysing procaine's response on porin expression, LB was prepared without NaCl and was supplemented with procaine (5 mM) when necessary. For analysing pH response on porin expression, LB without salt was buffered with 100 mM 2-(*N*-morpholino)ethanesulfonic acid or 100 mM 3-(N-morpholino)propanesulphonic acid, and pH was adjusted to 5.0 or 8.0, respectively, with HCl.

### DNA methods

Standard bacterial genetic methods were carried out as described by Silhavy *et al*. (1984). Deletion of genes from their chromosomal locations and subsequent curing of the antibiotic-resistant marker at the deletion sites were done using the λ-red mediated gene deletion method as described previously (Datsenko and Wanner, 2000). Deletions were confirmed using PCR. In some instances *lacZ* was recombined at the deletion site by the method of Ellermeier *et al*. (2002). Primers used for deletions and cloning are listed in the Table S2. Chromosomal fragments containing *envZ*, *ompR*, *cpxA*, *phoA* or *mzrA* were amplified using gene-specific cloning primers. Restricted PCR-amplified fragments were cloned into appropriately digested pBAD24, pBAD33 (Guzman *et al*., 1995), pTrc99A (GE Healthcare), pKT25, pUT18 or pUT18C (Karimova *et al*., 1998). The *mzrA*::*phoA* plasmid clone was created by PCR-amplifying *mzrA* fragments, restricting them with EcoRI and XbaI and ligating into predigested pBAD24. *phoA* fragments, digested with XbaI and SalI, were ligated into the appropriately digested pBAD24-*mzrA* (EcoRI-XbaI) clone. The Quickchange Site-Directed Kit from Stratagene was used to delete the putative TM domain from MzrA from the *mzrA-phoA* plasmid clone as well as to introduce the R397L mutation into the EnvZc clone as per the manufacturer's instructions using the primers listed in the Table S2.

### Protein methods

For Western blot analysis, overnight cultures were diluted 100-fold into appropriately supplemented media and grown 3 h. Cell pellets were resuspended in sample buffer and heated at 95°C for 5 min and analysed by SDS-PAGE. Urea (4 M) was added to the SDS-polyacrylamide running gel in order to better resolve OmpC and OmpF. Following electrophoresis proteins were transferred onto Immobilin-P (Millipore) using a mini-transblot (Bio-Rad) and incubated in primary antibody for 1.5 h. Primary rabbit antibodies and dilutions used were: OmpF/C/A (1:16 000), GroEL (1:50 000), LamB (1:10 000), MalE (1:10 000), FLAG (1:1000), TolC (1:5000). Goat anti-rabbit HRP-conjugated immunoglobulin G secondary antibodies were incubated for 1 h. Membranes were incubated with Immun-Star HRP substrate for 5 min and protein bands were visualized with a Molecular-Imager-ChemiDoc-XRS System from Bio-Rad. Protein bands were quantified using the Quantity One software from Bio-Rad. Primary mouse antibodies for DnaK (1:10 000) were incubated for 1.5 h followed by incubation with goat anti-mouse IgG alkaline phosphatase conjugate for 1 h. After 5 min exposure to enzyme-catalysed fluorescence substrate the membranes were scanned with a phosphorimager and analysed with the ImageQuant (Molecular Dynamics) program. For the identification of protein bands from SDS-PAGE gels, bands of interests were excised, and subjected to LC-MS/MS analysis at the University of Arizona Proteomics Consortium.

### EnvZc purification

EnvZc and EnvZcR397L were produced from plasmid pPH001, grown in LB and purified as previously described (Park and Inouye, 1997). The protein concentration was determined using a Micro BCA Assay kit from Pierce, using BSA standards.

### EnvZ phosphorylation and OmpR phosphotransfer assays

Phosphorylation and phosphotransfer experiments were carried out as described by Tran *et al*. (2000). Phosphorylation of EnvZ_c_ was performed in 20 μl reactions containing a (final concentration) of 50 mM Tris (pH 7.5), 50 mM KCl and 20 mM MgCl_2_. EnvZ_c_ and the EnvZ_c_ R397L were added at a final concentration of 4 μM, and the reactions were initiated by the addition of 2 μCi of [γ-^32^P]-ATP followed by incubation for 1, 2.5, 5 or 10 min at room temperature. For phosphotransfer reactions, after an initial 10 min autophosphorylation of EnvZ_c_ or EnvZ_c_ R397L, OmpR (2 μM final concentration) was added and incubation proceeded for 5, 15 or 30 min at room temperature. All reactions were stopped by the addition of 3 μl of denaturing sample buffer [124 mM Tris-HCl (pH 6.8), 20% (v/v) glycerol, 4% (w/v) SDS, 8% (v/v) β-mercaptoethanol and 0.025% (w/v) bromophenol blue]. Ten microlitres of each reaction was loaded on a 15% SDS-PAGE gel, and the gel was subsequently dried, exposed to a phosphorimager screen and visualized on a Molecular Dynamics Storm 860 Imager.

### Measurement of OmpR-dependent EnvZ_c_ ATPase activity

The EnvZ_c_-ATPase activity was assayed using a modification of the method of Brotherus *et al*. (1981) as described by Kenney (1997). Proteins were stored in a buffer containing 20 mM Tris, 0.1 mM EDTA, 750 mM NaCl and 0.9% glycerol at 4°C. The assay solution (pH of 7.2) was aliquoted to minimize freeze/thawing, and stored at −20°C. The reaction was performed in a total volume of 0.6 ml containing the following (mM): 0.5 EGTA, 130 NaCl, 20 KCl, 3 MgCl_2_, 3 Na_2_ATP, 50 imidazole and 0.03% albumin. EnvZ_c_ and OmpR were added at the following final concentrations: 2 and 1 μM respectively. The assay was initiated by the addition of the ATPase assay solution and stopped by the addition of 1 ml of a solution containing: 0.5% ammonium molybdate, 473 mM HCl, 1.5% SDS and 163 mM ascorbic acid. The samples were incubated for 10 min at 0°C, followed by the addition of 1.5 ml of a solution of 68 mM sodium citrate, 154 mM sodium arsenite and 2% glacial acetic acid and incubated at 37°C for 5 min. After 20 min at room temperature, the optical density of the samples at 850 nm was determined in a Beckman Coulter DU800 spectrophotometer and compared with Na_2_HPO_4_ standards. The ATPase activity was expressed as nanomoles of P_i_ liberated per ml and the nonenzymatic hydrolysis was corrected for by the inclusion of blanks that did not contain protein. EnvZ_c_ ATPase activity, which is independent of OmpR, was subtracted from the total ATPase activity when both proteins were present and the corrected values are indicated.

### Enzymatic assays

β-Galactosidase and alkaline phosphatase activities were determined by published methods (Miller, 1992; Michaelis *et al*., 1983). These activities were measured from culture grown to mid-log phase (OD_600_ of 0.7–0.9). Kinetic analysis of enzyme activity was carried out using a VersaMax (Molecular Dynamics) microtiter plate reader in quadruplicate.

### Cell fractionation

Overnight cultures were diluted 1:100 and grown 3 h with appropriate supplements. Equivalent amounts of cells, based on OD_600_, were pelleted and resuspended in a periplasmic extraction buffer (10 mM Tris-HCl pH 7.5, 0.5 M sucrose, 10 mM EDTA, 0.2 mg ml^−1^ lysozyme) and periplasm was extracted by the gentle osmotic shock method (Arié*et al*., 2001). Following extraction of the periplasm cells were pelleted and washed before resuspension in a lysis buffer (100 mM Tris pH 7.5, 10 mM MgCl_2_, 0.25 μg ml^−1^ DNase I and 2 mM PMSF) and lysed using a French Press. Cell lysates were centrifuged for 1 h at 105 000 *g*, 4°C to separate soluble (cytoplasm and residual periplasm) and insoluble (inner and outer membranes) fractions. Cell fractions were then subjected to SDS (urea)-PAGE (as described above) followed by Coomassie staining.

### Localization of MzrA-FLAG

Inner membrane proteins from envelopes of cells expressing MzrA-FLAG were solubilized at room temperature for 30 min in 0.3% solution of sarcosyl, and insoluble material was removed by centrifugation at 4°C for 1 h at 105 000 *g*. Cell envelopes were separated into inner and outer membranes by sucrose density gradients 30–55% (wt/vol) as described previously (Misra *et al*., 2000). Gradient fractions were subjected to SDS-PAGE, followed by Western blot analysis (see above) probing for FLAG and TolC. Buoyant densities were calculated by measuring the refractive index of each fraction at room temperature.
