# Supplementary material for: MzrA: a novel modulator of the EnvZ/OmpR two-component regulon
Source: Mol Microbiol. 2009 May 26;72(6):1408–22. doi: 10.1111/j.1365-2958.2009.06728.x (PMC2727453; doi:10.1111/j.1365-2958.2009.06728.x)
Supplement: Supplementary file 1 [file mmi0072-1408-SD1.pdf]

```
P42615     ----MQIPRMSLRQLAW---SGAVLLLVGTLLLAWSAVRQQESTLAIRAVHQGTTMPDGF  53   YQJB_ECOLI
Q3YXD0     ----MQIPRMSLRQLAW---SGTVLLLVGTLLLAWSAVRQQESTLAIRAVHQGTTMPDGF  53   Q3YXD0_SHISS
A8APZ0     ----MVKPHITLRQLAW---TTSFLVLMSVLFLVWSTIRQQESTLAIRAIHQGVSMPDGF  53   A8APZ0_CITK8
Q8XF92     ----MLKPRITARQLIW---ISAFLLMLTILMMTWSTLRQQESTLAIRAVNQGASMPDGF  53   Q8XF92_SALTI
A6TED5     ---MMVMKRPSLRQFSW---LLGGSLLLGALFWLWLAVQQQEATLAIRPVGQGIGMPDGF  54   A6TED5_KLEP7
A4WES8     ----MVISPLALRRLSY---GLIALVLLSALILVWTALQRQESTLAIRPVSQGASVPDGF  53   A4WES8_ENT38
A7MIS1     --------------------MLSAGALLAAMLLCWAALQKHETTLEIRAVSQGATIPDGF  40   A7MIS1_ENTS8
Q7CGC4     ----MINFRGRFGRPLWHYLVLPVVLLLLAVILLTPMIVQTESTLKIRPNQQGLSLPDGF  56   Q7CGC4_YERPE
Q6D9H4     MSIRWLSPTLSPRKIGR--ILLLLALPIIALTQSQPPRHSQDDAMLHIKPYDGAALPDGF  58   Q6D9H4_ERWCT
                                      :  :          : ::      :*  :****

P42615     SIWHHLDAHGIPFKSITPKNDTLLITFDSSDQSAAAKAVLDRTLPHGYIIAQQDNNSQAM  113  YQJB_ECOLI
Q3YXD0     SIWHHLDAHGIPFKSITPKNDTLLITFDSSDQSAAAKAVLDRTLPHGYIIAQQDNNSQAM  113  Q3YXD0_SHISS
A8APZ0     SIWHHLDANGIRFKSITPQNDALLITFDSSAQSAAAKAVLDRTLPHGYIIAQQNGDSQAV  113  A8APZ0_CITK8
Q8XF92     SVLHHLDANGIHFKSITPKNDMLLITFDSPAQSAAAKTVLDQTLPHGYVVAQQDDDNETV  113  Q8XF92_SALTI
A6TED5     SVWHHLDANGIRFKSITPQKDGLLIKFDSTAQGAAAKEVLGRALPHGYIIALLEDDNSPT  114  A6TED5_KLEP7
A4WES8     YIWHHLDANGIQFKSITPQDDVLLIKFDSSAQSAAAKVVLDRSLPRGYIIALQDDQSQTA  113  A4WES8_ENT38
A7MIS1     SVWHHLDANGIRFKSITPQDDILLIKFDTSDQSEAARKVLYRSLPRGFVIAQQEEKSLTP  100  A7MIS1_ENTS8
Q7CGC4     YLYQHLNGRGIHIKSIIPENDSLVVSLEFPEQQMQAIEVLQDVLPAGYAIVASESKKR-H  115  Q7CGC4_YERPE
Q6D9H4     YVYQRLNEKGIAKSITPEQDSLIVRLASPEQSIAARDVLRLSLPK-VTITAQQATSPTP  117  Q6D9H4_ERWCT
            : ::*: .** :*** *:.* *:: :  . *   *  **   **    :.  :  .

P42615     QWLTRLRDNSHRFG  127   YQJB_ECOLI
Q3YXD0     QWLTRLRDNSHRFG  127   Q3YXD0_SHISS
A8APZ0     QWLTRLRDNPHRFG  127   A8APZ0_CITK8
Q8XF92     QWLSRLRESSHRFG  127   Q8XF92_SALTI
A6TED5     AWLSRLRDAPHRLG  128   A6TED5_KLEP7
A4WES8     VWLTRLRDTSHRFG  127   A4WES8_ENT38
A7MIS1     AWLTRLRHDIQRLG  114   A7MIS1_ENTS8
Q7CGC4     RLLPVFRSNQQNLG  129   Q7CGC4_YERPE
Q6D9H4     FWQQKLTQKQSKLG  131   Q6D9H4_ERWCT
                :     .:*
```

Figure S1. Multiple sequence alignment of MzrA (YqjB) sequences from *Enterobacteriaceae*. Amino acid sequences were aligned using the CLUSTAL W (1.83) program (http://ch.EMBnet.org/). Amino acids that are either identical (*), have similar side chains (:) or similar overall characteristics (.) are shown in the last line.

Table S1: Bacterial strains and plasmids

Strain or Plasmid	Description	Reference or Source
MC4100	F- *araD139* Δ(argF-lac)U139 *rspL150 relA1 fibB5301 ptsF25 deoC1 thi*-1 *rbsR*	(Casadaban, 1976)
JM109	e14–(McrA–) *recA1 endA1 gyrA96 thi-1 hsdR17* ($r_K^- m_K^+$) *supE44 relA1* Δ(*lac-proAB*) [F′ *traD36 proAB* $lacI_qZ\Delta M15$].	Promega
RAM1292	MC4100 Δ*ara714*	(Werner and Misra, 2005)
BTH101	F- *cya99 araD139 galE15 galK16 rpsL1* (Str^r) *hsdR2 mcrA1 mcrB1*	EUROMEDEX
BL21(DE3)	F–, *omp*T, *hsd*S_B(r_B-, m_B-), *dcm*, *gal*, λ(DE3)	Novagen
RAM1318	MC4100 Δ*bamB*::scar *degP*::Tn*10*	(Charlson *et al*., 2006)
RAM1503	RAM1292 / pBAD24	This Study
RAM1504	RAM1292 / pBAD24-*mzrA*	This Study
RAM1505	RAM1292 / pBAD24-*mzrA*(FLAG)	This Study
RAM1506	RAM1292 / pBAD33	This Study
RAM1507	RAM1292 / pBAD33-*mzrA*(FLAG)	This Study
RAM1508	RAM1292 Δ*mzrA:*:scar	This Study
RAM1509	RAM1508/ pBAD33	This Study
RAM1510	RAM1508/ pBAD33-*mzrA*(FLAG)	This Study
RAM1511	RAM1508 Δ*omr*::*lacZ*	This Study
RAM1512	RAM1292 Δ*yqj*::*lacZ*	This Study
RAM1513	RAM1512 *zii*::Tn*10*	This Study
RAM1514	RAM1512 *cpxA** *zii*::Tn*10*	This Study
RAM1515	RAM1512 Δ*cpxR*::Cm^r *zii*::Tn*10*	This Study
RAM1516	RAM1292 *zii*::Tn*10*	This Study
RAM1517	RAM1292 *cpxA** *zii*::Tn*10*	This Study
RAM1518	RAM1516 Δ*mzrA*::scar	This Study
RAM1519	RAM1517 Δ*mzrA*::scar	This Study
RAM1520	RAM1516 Δ*envZ*::Cm^r	This Study
RAM1521	RAM1517 Δ*envZ*::Cm^r	This Study
RAM1522	RAM1292 Δ*omr*::*lacZ*	This Study
RAM1523	RAM1522 / pBAD24	This Study
RAM1524	RAM1522 / pBAD24-*mzrA*	This Study
RAM1525	RAM1522 zii::Tn*10*	This Study
RAM1526	RAM1522 *cpxA** *zii*::Tn*10*	This Study
RAM1527	RAM1522 Δ*cpxR*::Cm^r *zii*::Tn*10*	This Study
RAM1528	RAM1525 Δ*mzrA*::scar	This Study
RAM1529	RAM1526 Δ*mzrA*::scar	This Study
RAM1530	RAM1292 *ompR101*	This Study
RAM1531	RAM1530 / pTrc99A	This Study
RAM1532	RAM1530 / pTrc99A-*mzrA(*FLAG)	This Study
RAM1533	RAM1530 Δ*omr*::*lacZ*	This Study
RAM1534	RAM1292 *ompR472*	This Study
RAM1535	RAM1534 / pTrc99A	This Study
RAM1536	RAM1534 / pTrc99A-mzrA(FLAG)	This Study
RAM1537	RAM1292 Δ*envZ*::scar	This Study
RAM1538	RAM1537 / pTrc99A	This Study
RAM1539	RAM1537 / pTrc99A-mzrA(FLAG)	This Study
RAM1540	RAM1537 Δ*omr*::*lacZ*	This Study
RAM1541	RAM1292 *envZ*[R397L]	This Study
RAM1542	RAM1541 Δ*omr*::*lacZ*	This Study
RAM1543	BTH101 / pKT25 pUT18C	This Study
RAM1544	BTH101 / pKT25*mzrA* and pUT18C	This Study
RAM1545	BTH101 / pKT25*mzrA* and pUT18C*envZ*	This Study
RAM1546	BTH101 / pKT25*mzrA* and pUT18*envZ*	This Study

RAM1547	BTH101 / pKT25*mzrA* and pUT18C*ompR*	This Study
RAM1548	BTH101 / pKT25*mzrA* and pUT18*ompR*	This Study
RAM1549	BTH101 / pKT25*envZ* and pUT18*envZ*	This Study
RAM1551	RAM1292 Δ*ppiA*::*lacZ*	This Study
RAM1552	RAM1551/ pBAD24	This Study
RAM1553	RAM1551/ pBAD24-*mzrA*	This Study
RAM1554	RAM1292 Δ*ompF*::*lacZ*	This Study
RAM1555	RAM1554 / pBAD24	This Study
RAM1556	RAM1554 / pBAD24-*mzrA*	This Study
RAM1557	RAM1292 *mgrB*::λp*lac*Mu55	This Study
RAM1558	RAM1557 / pBAD24	This Study
RAM1559	RAM1557 / pBAD24-*mzrA*	This Study
RAM1560	RAM1292 *mgtA*::λp*lac*Mu55	This Study
RAM1561	RAM1560 / pBAD24	This Study
RAM1562	RAM1560 / pBAD24-*mzrA*	This Study
RAM1563	RAM1292 / pBAD24-*mzrA::phoA*	This Study
RAM1564	RAM1292 / pBAD24-*mzrA*[Δ13-28]*::phoA*	This Study
RAM1565	RAM1292 *dsbA*$^-$::Kan^r / pBAD24-*mzrA::phoA*	This Study
RAM1566	RAM1292 / pBAD24-*mzrA::lacZα*	This Study
RAM1567	RAM1292 / pBAD24-*mzrA*[Δ13-28]*::lacZα*	This Study
RAM1568	JM109 pBAD24	This Study
RAM1569	JM109 pBAD24-*mzrA*	This Study
RAM1570	JM109 pBAD24-*mzrA::lacZα*	This Study
RAM1571	JM109 pBAD24-*mzrA*[13-28]*:: lacZα*	This Study
RAM1572	RAM1540 / pBAD24	This Study
RAM1573	RAM1540 / pBAD24-*mzrA*	This Study
RAM1574	RAM1292 Δ*rseA*::scar	This Study
RAM1575	RAM1574 Δ*yqj*::*lacZ*	This Study
RAM1576	RAM1292/ pTrc99A	This Study
RAM1577	RAM1292/ pTrc99A-*mzrA*(FLAG)	This Study
RAM1578	RAM1318/ pBR322	This Study
RAM1579	RAM1318/ pBR322 *exuT'exuRyqjABCDE'*	This Study
RAM1580	RAM1292 λRS88 *cpxP*'-*lacZ* *nadA*::Tn*10*	This Study
RAM1581	RAM1580/ pBAD24	This Study
RAM1582	RAM1580/ pBAD24-*mzrA*(FLAG)	This Study
RAM1583	RAM1530 Δ*yqj*::*lacZ*	This Study
RAM1584	RAM1537 Δ*yqj*::*lacZ*	This Study
RAM1585	RAM1541 Δ*yqj*::*lacZ*	This Study
RAM1586	RAM1292 *envZ11*	This Study
RAM1587	RAM1586 Δ*yqj*::*lacZ*	This Study
**Plasmids**		
pBAD24	Amp^r; Expression vector; arabinose inducible	(Guzman *et al*., 1995)
pBAD33	Cm^r; Expression vector; arabinose inducible	(Guzman *et al*., 1995)
pTrc99A	Amp^r; Expression vector; IPTG inducible	GE Healthcare
pBAD33*mzrA*-FLAG	Cm^r; pBAD33 containing the *mzrA*(FLAG) gene	This Study
pBAD24*mzrA*	Amp^r; pBAD24 containing the *mzrA* gene	This Study
pBAD24*mzrA*-FLAG	Amp^r; pBAD24 containing the *mzrA*(FLAG) gene	This Study
pBAD24*mzrA*-*phoA*	Amp^r; pBAD24-*mzrA*::*phoA*	This Study
pBAD24*mzrA*[Δ13-28]-*phoA*	Amp^r; pBAD24-*mzrA*[Δ13-28]::*phoA*	This Study
pBAD24*mzrA*-*lacZα*	Amp^r; pBAD24-*mzrA*:: *lacZα*	This Study

pBAD24*mzrA*[Δ13-28]- *lacZα*	Amp[r]; pBAD24-*mzrA*[Δ13-28]::*lacZα*	This Study
pTrc99A *mzrA*-FLAG	Amp[r]; pTrc99A containing the *mzrA*(FLAG) gene	This Study
pKD3	Cm[r]; Plasmid carrying Cm[r] gene cassette	(Datsenko and Wanner, 2000)
pKD4	Kan[r]; Plasmid carrying Kan[r] gene cassette	(Datsenko and Wanner, 2000)
pKD46	Amp[r]; Red recombinase expression plasmid	(Datsenko and Wanner, 2000)
pCP20	Cm[r] and Amp[r]; FLP recombinase expression plasmid, temperature sensitive	(Datsenko and Wanner, 2000)
pKG136	*ahp* FRT *lacZY*[+] t_{his} oriR6K	(Ellermeier *et al*., 2002)
pKT25	Kan[r]; Harbors the T25 fragment of adenylate cyclase from *B. pertussis*	EUROMEDEX
pUT18	Amp[r]; Harbors the T18 fragment of adenylate cyclase from *B. pertussis*	EUROMEDEX
pUT18C	Amp[r]; Harbors the T18 fragment of adenylate cyclase from *B. pertussis*	EUROMEDEX
pKT25*mzrA*	Kan[r]; pKT25 with the *mzrA* gene	This Study
pKT25*envZ*	Kan[r]; pKT25 with the *envZ* gene	This Study
pKT25*ompR*	Kan[r]; pKT25 with the *ompR* gene	This Study
pUT18C*mzrA*	Amp[r]; pUT18C with the *mzrA* gene	This Study
pUT18C*envZ*	Amp[r]; pUT18C with the *envZ* gene	This Study
pUT18C*ompR*	Amp[r]; pUT18C with the *ompR* gene	This Study
pUT18C*cpxA*	Amp[r]; pUT18C with the *cpxA* gene	This Study
PUT18*envZ*	Amp[r]; pUT18 with the *envZ* gene	This Study
pUT18*ompR*	Amp[r]; pUT18 with the *ompR* gene	This Study
pUT18*cpxA*	Amp[r]; pUT18 with the *cpxA* gene	This Study
pBR322	Amp[r];Tet[r] cloning vector	(Bolivar, *et al.* 1977)
pBR322-*exuT' exuRyqjABCDE'*	Amp[r];pBR322 with *exuT'exuRyqjABCDE'* genes	This Study

1. Bolivar F, Rodriguez RL, Green PJ, Betlach MC, Heyneker HL, Boyer HW, Crosa JH, and Falkow S. (1977) Construction and characterization of new cloning vehicles. II. A multipurpose cloning system. *Gene* 2, 95–113.
2. Casadaban M J (1976) Transposition and fusion of the lac genes to select promoters in *Escherichia coli* using bacteriophage lambda and Mu. *J Mol Biol* **141:**541-555.
3. Charlson E S, Werner J N, Misra R (2006) Differential effects of *yfgL* mutation on the biogenesis of *Escherichia coli* outer membrane proteins and lipopolysaccharide. *J Bacteriol* **188:**7186-7194.
4. Datsenko K A, Wanner B L (2000) One-step inactivation of chromosomal genes in *Escherichia coli* K-12 using PCR products. *Proc Natl Acad Sci USA* **97:**6640-6645.
5. Ellermeier C D, Janakiraman A, Slauch J M (2002) Construction of targeted single copy *lac* fusions using λ Red and FLP-mediated site-specific recombination in bacteria. *Gene* **290:**153-161.
6. Guzman L M, Belin D, Carson M J, Beckwith J (1995) Tight regulation, modulation, and high-level expression by vectors containing the arabinose P_{BAD} promoter. *J Bacteriol* **177:**4121-4130.
7. Werner J, Misra R (2005) YaeT (Omp85) affects the assembly of lipid-dependent and lipid-independent outer membrane proteins of *Escherichia coli*. *Mol Microbiol* **57:**1450-1459.

Table S2: Primers used for cloning and deletion

Deletion Primers

Primer Name	Sequence (5'-3')
yqjA Fwd	GTTAACCGATTCACCAGGAATAATGAATGGAACTTTTGTGTAGGCTGGA GCTGCTTCG
mzrA Fwd	GAAATATGGAAATCGGGGGTAAGGGATGCAAATATGTAGGCTGGAGCT GCTTCG
mzrA Rev	GTGAGTGATATGTTTCGGATAACAGGAAGTTACATATGAATATCCTCCTT AG
envZ Fwd	CTTTGTACCGGACGGCTCTAAAGCATGAGGCGATTGCGCTTCTGTAGGCT GGAGCTGCTTCG
envZ Rev	ACCTTCGCCTCCCGTTTATTTACCCTTCTTTTGTCGTGCCCATATGAATAT CCTCCTTAG
omrB Fwd	CCGCTGGTGGCGTTTGGCTTCAGGTTGCTAAAGTGGTGATTGTAGGCTGG AGCTGCTTCG
omrA Rev	ACACGAGATAAAGAACGCGAGCGACAGTAAATTAGGTGCGCATATGAA TATCCTCCTTAG
cpxR Fwd	TTTCTGCCTCGGAGGTATTTAAACAATGAATAAAATCCTGTGTAGGCTGG AGCTGCTTCG
cpxR Rev	AGATGGCGAAGATGCGCGCGGTTAAGCTGCCTATCATGAAGCCATCATA TGAATATCCTCCTTAG
rseA Fwd	GGATACTGGATAAGGGTATTAGGCATGCAGAAATGTGTAGGCTGGAGCT GCTTCG
rseA Rev	CCAAAGTTGCTTCATTACTGCGATTGCGTTCCTAACATATGAATATCCTC CTTAG

Expression Vector Primers (restriction sites shown in lower case)

Primer Name	Sequence(5'-3')	Cloning Vector
*mzrA Eco*RI Fwd	AGGAGgaattcACCATGCAAATACCTCGC	pBAD24
*mzrA Xba*I Fwd	TGCAtctagaAATATGGAAATCGGGGGTAAGGGATGC AAATA	pBAD24 and pBAD33
*mzrA HinD*III Rev	CGATCaagcttGAGTGATATGTTTCGGATAAC	pBAD24
mzrA-FLAG *HinD*III Rev	CGATCaagcttACTTATCGTCGTCGTCCTTGTAGTCAC CGAAGCGATGAGAGTTATC	pBAD24 and pBAD33
*mzrA Xba*I Rev	CAGTtctagaTCCGAAGCGATGAGAGTTATC	pBAD24
*phoA Xba*I Fwd	ATAtctagaATGCCTGTTCTGGAAAACCGG	pBAD24*mzrA*
*phoA Sal*I Rev	ATATgtcgacTTATTTCAGCCCCAGAGCG	pBAD24*mzrA*

Two Hybrid System Primers (restriction sites shown in lower case)

Primer Name	Sequence (5'-3')	Cloning Vector
mzrA E2HS *Xba*I Fwd	AGCTGtctagaGCAAATACCTCGCATGTCGC	pUT18C and pKT25
mzrA E2HS *Kpn*I Rev	GCAGTggtaccCCGAAGCGATGAGAGTTATCCCG	pUT18C and pKT25
envZ E2HS *Xba*I Fwd	AGCTGtctagacAGGAGGTTGCGCTTCTCGCCACG	pUT18, pUT18C and pKT25
envZ E2HS *Kpn*I Rev	GCAGTggtaccCCTTCTTTTGTCGTGCCCTGC	pUT18, pUT18C and pKT25
ompR E2HS *Xba*I Fwd	AGCTGtctagaGCAAGAGAACTACAAGATTCTGG	pUT18, pUT18C
cpxA E2HS *Xba*I Fwd	ACGTGtctagaGATAGGCAGCTTAACCGCGCG	pUT18, pUT18C, and pKNT25
cpxA E2HS *Kpn*I Fwd	GCAGTggtaccCGACTCCtCTTATACAGCGGC	pUT18, pUT18C, and pKNT25

Site-Directed Mutagenesis Primers (alterations shown in lower case)

Primer Name	Sequence (5'-3')	Cloning Vector
mzrA Δ13-28 SDM Fwd	CGCATGTCGCTTCGCCAGCTAGCCTCCGCGGTTCG CCAGCAAGAGTC	pBAD24*mzrA::phoA,* pBAD24*mzrA::lacZα*
mzrA Δ13-28 SDM Rev	GACTCTTGCTGGCGAACCGCGGAGGCTAGCTGGCG AAGCGACATGCG	pBAD24*mzrA::phoA,* pBAD24*mzrA::lacZα*
EnvZ R397L Fwd	GCGGCGACAGcGCaCtCACCATTAGCGGCACGGGAT	pETEnvZc

EnvZ R397L Rev	TAGGG CCCTAATCCCGTGCCGCTAATGGTGaGtGCgCTGTC GCCGC	pETEnvZc
